# Supplementary material for: Short-term prediction of COPD exacerbations based on wearable vital sign monitoring
Source: PLOS Digit Health. 2026 May 28;5(5):e0001405. doi: 10.1371/journal.pdig.0001405 (PMC13218495; doi:10.1371/journal.pdig.0001405)
Supplement: S1 Text — (DOCX) [file pdig.0001405.s001.docx]

## S1 Text. Mathematical details of the BVS^3^ score computation

To ensure reliable analysis, several preprocessing steps were applied to the vital sign times series collected by the Bora band^®^. These steps aimed to handle missing values, reduce noise and standardize the data according to the patient baseline, while maintaining the physiological relevance of the signals.

Vital sign time series were first resampled from a frequency of every 10 minutes to once per hour using a median filter to mitigate possible noise from the vital sign estimation algorithm and natural noise inherent to the patient’s activity. The median filter was chosen for its robustness against outliers. To ensure consistent dynamics across all vital signs prior to an AECOPD event, we considered the desaturation (100 - SpO_2_) rather than SpO_2_ directly.

$$D_{O_{2}}=100-S_{p}O_{2} (1)$$

A Box-Cox transformation was applied to the vital sign data to adjust their distribution, which deviated from a Gaussian distribution, making it more close to normal distribution.

$$BC\left( y, \lambda\right)=\left\{ \begin{matrix} \frac{y^{\lambda}-1}{\lambda}if \lambda\neq0 \\ \log\left( y \right)if \lambda=0 \end{matrix} \right. (2)$$

The next step of data processing was Gaussian Process filtering of our vital signs individual time series using a Matern kernel. GPs are a powerful tool for smoothing and interpreting time series data. They help reduce noise and reveal underlying trends by modeling data in a flexible, probabilistic way. GPs can also interpolate missing values, ensuring a more continuous and reliable representation of the data without imposing a fixed mathematical model.

To mitigate high-frequency noise and variations associated with the circadian cycle and physical activity, a fourth-order low-pass filter with a cutoff frequency of 1 day^-1^ was applied to each time series.

The preprocessing results in three filtered time series, one for each vital sign, preserving meaningful patterns. The processed data is evenly sampled at one-hour intervals with no missing values.

By convention, we note the aggregated time series of vital signs with the following notation:

$$X^{t}=\left( HR^{t}, BR^{t}, D_{O_{2}}^{t} \right)$$

where t is the time considered during the follow-up of the patient at the rate of a value every hour, with therefore:

$$\forall t, X^{t}\in\mathbb{R}^{3}$$

Z-score time series ($Z^{t})$ were derived from each GP-filtered vital sign series, using the patient’s baseline, estimated via rolling mean ($\bar{X^{t}}$) and standard deviation filters ($\sigma_{lt}$) calculated over the preceding 15 days, to quantify deviations in a patient-specific manner:

$$\forall t,Z^{t}=\frac{X^{t}-\bar{X^{t}}}{\sigma_{lt}(X^{t})} (3)$$

Finally, the three vital sign Z-scores were integrated using the survival function of the normal distribution sf, as described by the following equation:

$$sf\left( x \right)=\frac{1}{\sqrt{2\pi}}\int_{x}^{\infty} e^{-t^{2}/2} dt (4)$$

$$BVS^{3}(t)=-\log\left( sf\left[ 2\left( \frac{Z_{HR}\left( t \right)+Z_{BR}\left( t \right)+Z_{SpO_{2}}\left( t \right)}{3} \right) \right] \right) (5)$$
