# Supplementary material for: Short-term prediction of COPD exacerbations based on wearable vital sign monitoring
Source: PLOS Digit Health. 2026 May 28;5(5):e0001405. doi: 10.1371/journal.pdig.0001405 (PMC13218495; doi:10.1371/journal.pdig.0001405)
Supplement: S1 Fig — (DOCX) [file pdig.0001405.s002.docx]

## S1 Fig. Window size analysis

In the main manuscript, we discuss the clinical considerations motivating the choice of a 10-day window. Here, we provide a supplementary analysis to verify that this choice was not driven by performance optimization and that alternative window sizes yield comparable performance.

We evaluated BVS^3^ performance on the same study population using alternative window sizes of 7 and 14 days. Figure 1 shows the corresponding ROC curves. No meaningful differences were observed, as reflected by comparable AUC values across all window lengths.
