# Supplementary material for: Short-term prediction of COPD exacerbations based on wearable vital sign monitoring
Source: PLOS Digit Health. 2026 May 28;5(5):e0001405. doi: 10.1371/journal.pdig.0001405 (PMC13218495; doi:10.1371/journal.pdig.0001405)
Supplement: S2 Text — (DOCX) [file pdig.0001405.s003.docx]

## S2 Text. BVS^3^ false positive analysis

A complete analysis of all false alerts raised by the BVS^3^ score would be most insightful but requires complete access to the patient’s medical records during their whole follow-up, which we unfortunately lack. However, we did perform a summary analysis of these false positives under the scope of the ROME proposal guidelines for exacerbation severity grading. These require for an exacerbation to be moderate for at least 3 of the following 5 criteria to be met:

- Dyspnea VAS ≥ 5
- HR ≥ 95 bpm
- RR ≥ 24 breaths/min
- Resting SpO₂ ≤ 92% AND/OR change ≥3%
- CRP ≥ 10mg/L

While patient-reported outcomes (VAS) and inflammatory markers (e.g., CRP) were not available, we were able to assess whether vital sign–based criteria were met. Resting SpO₂ was approximated using the median nightly value, while to limit outlier influence, the 75th diurnal percentile was used for respiratory and heart rates. Using these definitions, 16% of BVS^3^ false-positive alerts met all three vital sign criteria, suggesting that at least this proportion could have been classified as moderate or severe exacerbations under the ROME proposal. Additionally, 61% met at least two criteria and 88% met at least one. These findings suggest that many BVS^3^ alerts coincide with physiologic changes consistent with exacerbation-related events, even in the absence of complete clinical data.
