# Supplementary material for: Short-term prediction of COPD exacerbations based on wearable vital sign monitoring
Source: PLOS Digit Health. 2026 May 28;5(5):e0001405. doi: 10.1371/journal.pdig.0001405 (PMC13218495; doi:10.1371/journal.pdig.0001405)
Supplement: S1 Table — (DOCX) [file pdig.0001405.s004.docx]

## S1 Table. Detailed comparison with prior work for short-term prediction of AECOPD

| **Key limitations** | **Monocentric validation; multicentric prospective validation ongoing** | Self-reported AECOPD not confirmed by EHR or clinicians; data leakage risk due to absence of train/test split for cases raising concerns about limited external generalizability. | Self-reported AECOPD not confirmed by EHR or clinicians; potential criterion contamination as symptom questionnaires are used both to define mild AECOPD and as input features for prediction. | Self-reported AECOPD not confirmed by EHR or clinicians; study potentially dominated by mild exacerbations. | High risk of overfitting and dimensionality issues; model complexity (multi-task neural network with 153 features) substantially exceeds the number of severe events (55), raising concerns about limited external generalizability. |
| --- | --- | --- | --- | --- | --- |
| **Performance  (AUC, sensitivity at 15% FPR)** | **AUC 0.88 Sens@15% FPR = 74%** | AUC 0.73 Sens@15% FPR ≈ 40% | AUC 0.73 Sens@15% FPR ≈ 42% | AUC 0.68 Sens@15% FPR ≈ 39% | Severe AECOPD :  AUC 0.74 Sens@15% FPR ≈ 38% OCS initiation :  AUC 0.765 Sensitiviy not available |
| **Model & validation** | **Unsupervised statistical algorithm, validation cohort** | RF, partial train/test split (controls only), no external validation | EasyEnsemble classifier, Train/test split, no external validation | Logistic classifier, 10-fold cross-validation, no external validation | Multi-task neural net (MTNN), no external validation |
| **Sample size**  **(patients / cases / controls)** | **220 patients / 42 cases (35 moderate, 7 severe) / 2 536 controls** | 2 374 patients / 5 906 cases / Training : 49 122 controls Testing : 13 111 controls | 505 patients / Training : 869 cases / 38 561 controls Testing : 394 cases / 13 505 controls | 110 patients / 286 cases / 1005 controls | 135 patients / Severe AECOPD :  55 cases / 57 150 controls OCS initiations :  316 cases / 13 503 controls |
| **Data sources** | **Single passive wearable** | Symptom questionnaire on smartphone app | Symptom questionnaire on smartphone app | Pulse oximeter | Smartphone app, pulse oximeter |
| **Predicted outcome** | **Clinician-defined moderate/severe AECOPD (EHR-confirmed)** | Self-reported moderate/severe AECOPD | Self-reported moderate/severe AECOPD | Self-reported mild, moderate and severe AECOPD | Hospital admission (EHR-based), initiation of oral corticosteroid treatment |
| **Prediction horizon  (Time period)** | **10 days** | 3 days | 14 days | 7 days | 1 day |
| **Study** | **BVS3 (this study)** | Chmiel et al., 2022 | Glyde et al., 2023 | Shah et al., 2017 | Orchard et al., 2018 |

| **Key limitations** | Study dominated by mild exacerbations; potential criterion contamination as symptom questionnaires are used both to define mild AECOPD and as input features for prediction. | Unclear AECOPD definition; high risk of overfitting due to a very low event-to-feature ratio (25 events, 45 features) and the use of a deep neural network, which may limit generalizability. | Unclear AECOPD definition; incomplete reporting of event counts used for model training, preventing assessment of overfitting risk and model robustness. | Case–control ratio does not reflect real-world daily prevalence, unclear selection strategy of controls likely yielding optimistic performance estimates. | High patient burden limiting acceptability; unclear AECOPD severity distribution with likely dominance of mild AECOPD; potential criterion contamination as symptom questionnaires are used both to define mild AECOPD and as input features for prediction. |
| --- | --- | --- | --- | --- | --- |
| **Performance  (AUC, sensitivity at 15% FPR)** | AUC not computed Sens@16% FPR ≈ 98% | AUC 0.985 Sens@15% FPR ≈ 95% | Validation cohort :  AUC and ROC not reported Sens@19%FPR = 69% | AUC 0.74 Sens@61%FPR = 99% | AUC 0.87 Sens@21%FPR = 85% |
| **Model & validation** | Validation cohort | DNN, no external validation | RF, validation cohort | RF, validation cohort | RF, 10-fold cross-validation, no external validation |
| **Sample size (patients / cases / controls)** | 90 patients / 112 clinician defined AECOPD (4 severe, 108 mild/moderate with 14 requiring medication escalation) / 291 cases / 2 860 controls | 67 patients / 25 cases / ~ 5 600 controls | 177 patients / Training : 140 patients /  External validation : 39 patients Number of exacerbations not indicated | External validation :  2 151 patient / 3 639 cases / 6 048 controls | 101 patients / 1 714 cases / 8 800 controls |
| **Data sources** | Symptom questionnaire on smartphone app, spirometry, CRP | smartphone app, wearable, home air quality sensing device, environmental open data | smartphone app, wearable, home air quality sensing device, environmental open data | EHR, spirometry, environment | Smartphone app, wearable air quality sensor, spirometer |
| **Predicted outcome** | Self-reported mild, moderate and severe AECOPD confirmed by clinician | AECOPD, unclear definition | AECOPD, unclear definition | Moderate and severe AECOPD (EHR-based) | Self-reported mild, moderate and severe AECOPD confirmed by clinician |
| **Prediction horizon  (Time period)** | 14 days | 7 days | 7 days | 1 day | 1 day |
| **Study** | Patel et al., 2021 | Wu et al., 2021 | Wu et al., 2022 | Jo et al., 2023 | Atzeni et al., 2025 |
