# Supplementary material for: Short-term prediction of COPD exacerbations based on wearable vital sign monitoring
Source: PLOS Digit Health. 2026 May 28;5(5):e0001405. doi: 10.1371/journal.pdig.0001405 (PMC13218495; doi:10.1371/journal.pdig.0001405)
Supplement: Attachment — Submitted filename: Response to Reviewers 2026-02-14.docx [file pdig.0001405.s005.docx]

# Reviewer #1

This paper proposes an approach to detect early detection of acute exacerbations of chronic obstructive pulmonary disease using remote patient monitoring. The developed method has been evaluated using a collected dataset to validate its performance. The study is important; however, some details of the study could be further elaborated.

Line 17: Provide the full name of BVS3, as this is the first time this abbreviation appears.

Thank you for your remark, we added the full name of BVS^3^ (Bora Vital Sign Standard Score) in the manuscript.

Line 40:advanced multi-sensor systems", please name examples of such systems.

We have revised the manuscript to explicitly name two examples of advanced multi-sensor systems that have reported high predictive performance, citing approaches described by Wu and colleagues and Atzeni and colleagues.

Line 82: The BVS3 score was already developed in [21] by the authors? If so, then it cannot claim the development of this score as part of the contributions of this paper. Please clarify.

Reference [21] is a patent that describes a general methodological framework for predicting COPD exacerbations. The patent does not name or define the BVS3 score, nor does it include a concrete instantiation of the score or any clinical performance evaluation.

In the present manuscript, we introduce BVS3 as a specific instantiation of this broader framework and provide its full scientific description, including score construction and comprehensive clinical validation on patient data. We have revised the manuscript to clarify this distinction and to ensure that the contribution claims accurately reflect this scope.

**Changes** in the manuscript:

In this context, we previously **described a methodological framework for risk estimation in a patent [21]. In the present work, we introduce the Bora Vital Sign Standard Score (BVS^3^), a specific instantiation of this risk score framework** derived using machine learning techniques applied to vital signs data (heart rate, breathing rate, SpO₂) collected through RPM via a connected wristband.

Line 109: "It is an unsupervised algorithm that combines predefined mathematical formulas with machine learning". I didn't see any machine learning component (e.g., classification model) in the calculation of BBS3 scores. It is fully determined by the statistics, so it is a statistical approach. Therefore, it might not be accurate to call it a machine learning approach.

We agree that describing BVS3 as a “machine learning score” may be misleading, as the learning (Gaussian process for processing time series) is performed on the signal structure, and there is no training on the outcomes (exacerbations dates). As such, the BVS3 score can be seen as an unsupervised statistical model rather than a machine learning one. We have therefore revised the title to “Short-term prediction of COPD exacerbations based on wearable vital signs monitoring” and updated the abstract and manuscript text accordingly.

Line 169: HR, BR, and SpO₂ are used in the proposed approach. Besides the practical consideration of continuous measurement, are there any other justifications (from the machine learning perspective, such as feature importance) for the choice of these signals?

Our approach builds on the expert-driven feature selection performed in the Rome proposal. In that work, a panel of experts evaluated 21 candidate variables identified through a comprehensive literature review within a conceptual framework describing the causes, pathobiological mechanisms, and physiological consequences of AECOPD. Through consensus, dyspnea, heart rate, respiratory rate, oxygen saturation, and serum CRP were selected to grade mild and moderate AECOPD, with arterial blood gas analysis (PaCO₂ and pH) required to classify severe events.

We deliberately adopted this knowledge-driven ranking as an a priori feature selection step. We have revised the manuscript to explicitly introduce this rationale before discussing practical considerations related to continuous monitoring.

Line 183: "Gaussian Process filtering was applied to denoise, interpolate missing values." The hyperparameter of the filtering should be documented, such as the sliding window size.

Thank you for pointing that out. Gaussian process filtering with a Matérn kernel was applied using a 30-day sliding window, with hyperparameters optimized by maximizing the log-likelihood, to denoise the vital sign time series and interpolate missing values. We updated the manuscript accordingly.

Line 210: It would be good to present two figures to visually compare the BVS3 score in two scenarios (normal vs. alert).

We have updated Figure 3 to better highlight the normal period (D0‑30 to D0‑10) by shading it in light green, enabling clearer visual comparison with the alert period and facilitating interpretation of BVS3 score dynamics.

Line 312: "Robustness was further strengthened by incorporating temporal filtering to mitigate effects of short-term variations." It would be good to show how many false alerts were reduced.

We have revised the Discussion section to provide additional insight into the effects of temporal filtering. To sum up: AUC goes from 0.84 to 0.88 and specificity at 15% false positive rate from 69% to 74%.

Line 325: It is very important to compare with the previous study. Please clarify the details, such as the experimental setup. Also, it would be good to use a table to compare the performance.

Thank you for this important comment. We have substantially expanded the comparison with prior studies by adding a detailed table in the Supplementary Materials (Supplementary Table S1) that summarizes the experimental setup of previous short-term AECOPD prediction studies, including prediction outcomes and horizons, data sources, performance metrics, and key limitations. In addition, we have included a concise summary table in the Discussion section (Table 3) to facilitate direct comparison of predictive performance and methodological characteristics across studies.

# Reviewer #2

Thank you for submitting this manuscript examining the BVS3 score for predicting COPD exacerbations using wearable vital sign monitoring. The work addresses a clinically meaningful problem and demonstrates promising feasibility data. However, several issues require attention.

Major issues:
1. The characterization of BVS3 as a "machine learning score" is imprecise and potentially misleading. The algorithm employs predefined mathematical formulas with Gaussian process signal processing for data interpolation, not supervised learning trained on outcomes. Please revise the title, abstract, and throughout to accurately describe the methodology (e.g., "algorithm-based" or "signal-processing-based" risk score).

We agree that describing BVS3 as a “machine learning score” may be misleading, as the learning is performed on the signal structure rather than on outcomes. As such, the BVS3 score can indeed be seen as an unsupervised statistical model rather than a machine learning one. We have therefore revised the title to “Short-term prediction of COPD exacerbations based on wearable vital signs monitoring” and updated the abstract and manuscript text accordingly.

2. The paper has 42 AECOPD events with "no missing remote monitoring data." Please clarify: How many total AECOPD events occurred during the study period? What proportion were excluded due to missing data, and did excluded patients differ systematically from included patients? This information is essential for assessing generalizability. Maybe I missed it, but I re-read again trying to find, even if it said 86% followed the protocol.

Thank you for your comment. During the study period, a total of 58 AECOPD events, all validated by the clinical team, were documented in 51 patients at Verdun’s Hospital. Of these, 16 events were excluded from the analysis: 4 events were detected by the BVS3 score during the dismissed period and were excluded to avoid uncertainty about whether the detection was related to the exacerbation or to an unrelated physiological change, and 12 events were excluded because the total number of vital sign measurements during the 10-day case period was below 60 (either due to a broken sensor, or patients that stopped wearing the device), which was considered insufficient to reliably evaluate the algorithm’s performance.

This resulted in 42 AECOPD events (7 severe and 35 moderate) in 39 patients with complete remote monitoring data included in the analysis. Comparative analysis showed no statistically significant differences in baseline characteristics between patients with excluded events (number of patients=12) and those with included events, suggesting that the exclusions were not systematically related to patient characteristics or disease severity and are therefore unlikely to impact the generalizability of our findings.

We have updated the manuscript to clarify the total number of AECOPD events, the proportion excluded, the reasons for exclusion, and the absence of systematic differences between excluded and included patients.

3. Sample size concerns - this is different from missing data above. The severe AECOPD subgroup (n=7) is too small for reliable inference. While acknowledging this limitation is appreciated, consider removing or substantially downweighting the severe-specific analyses, or present these findings explicitly as exploratory with wider confidence intervals.

We agree that the analysis of severe AECOPD events (n = 7) is exploratory due to the small sample size. We therefore have revised the Results section to downweight the emphasis on severe events and explicitly reporting n = 7 alongside the performance metrics to provide context. In addition, we removed in the Discussion section the mention to the BVS3 performance on severe AECOPD (Line 270) to avoid overinterpretation.

4. The manuscript would benefit from direct comparison with existing validated prediction tools. Could you apply the ACCEPT tool or similar established models to your cohort to provide benchmarking context?

We considered comparing BVS3 with existing validated prediction tools. However, the ACCEPT tool predicts the annual exacerbation rate based on patient characteristics (age, sex, BMI, smoking status, domiciliary oxygen therapy, lung function, symptom burden, and current medication) and is designed to guide long-term treatment personalization. In contrast, BVS3 predicts exacerbation occurrence within the next 10 days and is intended for proactive remote monitoring, enabling nurses to intervene immediately in response to alerts. Because the objectives, prediction horizons, and input data differ fundamentally, a direct comparison with ACCEPT is not meaningful.

The Rome proposal provides a severity classification of AECOPD based on absolute thresholds for dyspnea, respiratory rate, heart rate, SpO₂, CRP, and arterial blood gases. While conceptually closer, our dataset lacks some of the required variables (dyspnea VAS, CRP and arterial blood gases), preventing a fully comparable implementation.

Other short-term predictive tools are either proprietary or rely on data types not available in our cohort (questionnaires, spirometry, etc.). The NEWS (National Early Warning Score) could in principle be applied, but we lack some required input data (systolic blood pressure, temperature, AVPU score), and it is not disease-specific, so any comparison would be limited.

To address the need for direct comparison, we have substantially expanded the comparison with prior studies by adding a detailed table in the Supplementary Materials (Supplementary Table S1) that summarizes the experimental setup of previous short-term AECOPD prediction studies, including prediction outcomes and horizons, data sources, performance metrics, and key limitations. In addition, we have included a concise summary table in the Discussion section (Table 3) to facilitate direct comparison of predictive performance and methodological characteristics across studies.

5. False-positive analysis: Please provide more detailed characterization of false positive alerts. What clinical events or circumstances triggered false alarms? This information is critical for understanding clinical workflow implications.

Although detailed characterization of the score’s false positives would be highly informative, such analysis requires complete access to patients’ medical records throughout the study period, which was not available. We therefore added a summary analysis comparing false-positive alerts to the ROME/GOLD exacerbation grading criteria. Specifically, 88% of false-positive alerts met at least one resting vital sign criterion for a moderate-to-severe exacerbation, and 16% met three criteria (which is the minimum required by the ROME proposal for exacerbation gradation). This suggests some of our false positives might reflect unrecorded clinically significant events. A supplementary note called “3. False positives analysis” has been added to the Supplementary Materials. This analysis is intended to provide physiological context to the alerts and does not imply reclassification of FP events as confirmed exacerbations. The assessment of their clinical decision-making value and workflow impact will require dedicated prospective interventional studies.

Minor issues:
6. Table 2 formatting: The "delta vs BVS3" column shows percentage point differences in sensitivity but could be misinterpreted. Please clarify these are absolute differences, not relative changes.

We have added a footnote to Table 2 to clarify the meaning of the “Delta* vs BVS3” column:

*“Absolute difference in sensitivity.”

This ensures that the values are clearly interpreted as absolute differences rather than relative changes. No changes were made to the column heading to maintain consistency with the original table format.

7. Please specify the bootstrap resampling parameters (number of iterations, resampling strategy) used for confidence interval estimation.

We have updated the Methods section to include details of the bootstrap procedure:

“Confidence intervals for the AUC and other performance metrics were estimated using a stratified, non-parametric percentile bootstrap with 1000 resampling iterations.”

8. Threshold selection - The threshold of 3 was chosen based on "preliminary data" for 85% specificity. Please provide more detail on this calibration dataset and whether any overfitting concerns exist.

Thank you for pointing out the need for clarification.

The threshold was not calibrated or optimized using a separate dataset. Instead, discrimination of case and control periods by the BVS^3^ score was first evaluated across the full range of decision thresholds using ROC analysis on the study dataset. A target specificity of 85% (false positive rate of 15%) was defined a priori based on anticipated clinical deployment constraints, corresponding to an acceptable alert burden in routine use, corresponding in our case to 5-6 alerts per year per patient considering the 10-day prediction horizon and the annual prevalence of AECOPD (1-2 AECOPD/year).

The numeric threshold value (BVS^3^ = 3) corresponds to the score yielding this predefined operating point on the ROC curve.

Importantly, the alert threshold was not selected to maximize outcome-based performance metrics, and no data-driven optimization or calibration was performed. Because threshold selection was based solely on a fixed specificity criterion applied after ROC construction, this approach minimizes overfitting concerns associated with threshold tuning. We have updated the Introduction and Methods section accordingly to clarify this point.

9. While COI disclosures are provided, please add a statement in the Discussion explicitly acknowledging how industry involvement was managed to ensure scientific integrity.

We have added the following statement in the Discussion section to clarify this point:

*“Finally, the involvement of a research team at Biosency introduces the possibility of institutional bias. Although this was addressed through use of the independent academic MARS dataset (HP2 laboratory), in which all acute exacerbation dates and vital-sign time series were validated and locked before analysis, and by prespecified, quantitative development and evaluation of the score without post-hoc optimization with respect to AECOPD event dates, further validation by independent groups will be important to confirm generalizability.”*

# Reviewer #3

Comments on COPD paper D-25-01009

This is overall an interesting and well-constructed paper on an important topic appropriate to PLOS Digital Health. I provide comments below. Some are not entirely trivial. However, I expect that they can all be addressed without undue difficulty by the authors. Also, AI believe that carefully addressing these questions will substantially boost the impact of the paper. Thanks for this opportunity to read about an interesting and useful study.

(line number : comment. The most important comments are preceded by **.)

17 - 18 : Perhaps clarify this sentence: "this study has two components. It introduces ... , and also retrospectively..." Or something like this. The sentence is not clear as-is.

We modified the manuscript to improve clarity: “ *This study proceeds in two steps : we first introduces the Bora Vital Sign Standard Score (BVS3), a novel machine learning score that predicts AECOPD using vital signs collected at home through remote patient monitoring, and then retrospectively evaluates its predictive performance in identifying AECOPD events ahead of clinician-defined episodes.”*

** 25 - 26 : (this comment comes up multiple times) What is the connection/relevance of these results to clinical use cases?

The aim of this study was to evaluate the predictive performance and anticipation capability of the BVS3 score rather than to demonstrate clinical benefit. We have clarified in the manuscript that the intended clinical relevance of BVS3 lies in its role as an early warning or decision-support tool within remote patient monitoring programs, designed to prompt earlier clinical review or intensified monitoring. We have intentionally avoided claims regarding improved outcomes, as these would require prospective interventional validation.

75 : Perhaps you can provide a more general context, with citations, of wearables for clinical use cases (Sibel, Oura, etc). Then narrow down to wearables for COPD.

Thank you for your comment. We have revised the Introduction to provide a broader context on wearable technologies and their clinical use, highlighting their validated application for continuous, non-invasive monitoring and early detection of clinical deterioration across multiple care pathways, including perioperative surveillance, infectious diseases, and heart failure. Relevant references have been added to support the general clinical applicability of wearable platforms prior to narrowing the discussion to their use in COPD and AECOPD prediction.

** 92 - 95 : (this comment comes up multiple times) Please discuss what clinicians want - any ML or automated intervention should be placed in this context. For example, if clinicians want automatic warnings of potential AECOPD, what sensitivity do they need? Do they need warnings for mild exacerbations (mentioned in Rome, not covered in this paper)? What is their tolerance for "alert fatigue" (in this paper, 85% specificity would give, on this cohort, 400 false alarms for 20 - 30 true alarms; is this acceptable?)

We have revised the Introduction to explicitly situate the evaluation of the BVS3 score within clinicians’ expectations and operational constraints.

First, we now clarify that clinicians primarily prioritize the early identification of moderate to severe AECOPD, which represent clinically actionable events requiring therapeutic intervention clinicians in accordance with current GOLD recommendations. Mild exacerbations, although clinically relevant, were outside the scope of the present study because the intended clinical action differs: these events are typically managed by patients themselves through self-administered therapy, whereas moderate and severe exacerbations require intervention by healthcare professionals. Accordingly, the BVS3 score evaluated here is intended for professional decision support and should be considered a separate device from any system designed to detect mild exacerbations.

Second, we now explicitly frame alert burden as the dominant constraint for short-term AECOPD detection. AECOPD are rare events, occurring on average one to two times per patient per year, and predictions are evaluated daily. Under these conditions, the absolute number of false alerts will necessarily exceed the number of true alerts, even for models with high sensitivity. Clinical acceptability therefore depends primarily on maintaining a sustainable alert frequency for healthcare professionals rather than on maximizing sensitivity alone.

On this basis, we justify the choice of a 15% false-positive rate by citing prior remote patient monitoring studies suggesting that approximately five to ten alerts per patient per year represents a tolerable alert burden for healthcare professionals. The selected operating point, corresponding to approximately six alerts per patient per year in our cohort, was intentionally conservative and prespecified to reflect realistic clinical deployment scenarios.

Finally, we added a summary analysis of BVS3 false-positive alerts in the Supplementary Materials to contextualize their clinical relevance. Although the operating point selected for BVS3 yields a relatively high number of false positives, some of these alerts appear to be associated with physiologic changes consistent with clinically relevant events for pulmonologists, and in some cases may correspond to unlabeled exacerbations.

** 101, 125 : (this comment comes up multiple times) You note that the wearable

device recorded physical activity. Why was this information not used? This is relevant because physical activity affects HR, BR, and maybe SpO2, and the Rome protocols specify resting rates of HR and BR. Also, does sleep represent a distinct type of activity with its own AECOPD diagnosis rubric? Sleep can be likely be inferred from the device readings, bolstered by a bayesian prior that it occurs at night.

This represents an interesting idea for improving the score. Activity data were not incorporated in the present study because the device did not provide activity measurements at a temporal resolution directly aligned with the vital sign recordings, and normalization of vital signs using activity signals felt nontrivial and a work in itself. We do, however, intend to investigate activity-informed normalization strategies in future work.

With respect to sleep, no validated sleep detection model was available for this device. Such an approach could be beneficial, and we are indeed working on simplified modelizations to begin with. In the current framework, removal of circadian effects via temporal filtering was intended to mitigate day–night dependence in the vital signs.

103 : What is the impact of not having controls (ie healthy people)? Normally, controls are a valuable resource to set baseline or noise levels. This paper uses time periods distant from a clinically-recorded AECOPD episode. Is this an adequate set of controls? (I don't know, but the question certainly comes up for the reader)

Thank you for raising this important point. Indeed, the absence of healthy controls prevents us from characterizing the behavior of the score in individuals without COPD, and therefore from estimating a false positive rate in a healthy population. This is a limitation of the present dataset.

However, the proposed score is not intended as a screening or diagnostic tool for the general population, but as a longitudinal risk monitoring tool deployed exclusively in patients with established COPD during long-term remote patient monitoring (RPM). In this context, the clinically relevant question is not how the score behaves in healthy individuals, but how often it generates alerts during periods in which no acute exacerbation of COPD (AECOPD) is clinically observed.

For this reason, periods temporally distant from clinically recorded AECOPD episodes were used as control periods. These periods correspond to real-world “stable COPD” conditions during which an alert would be considered a false positive in clinical practice, as it would trigger unnecessary attention from healthcare professionals. Using these periods as controls therefore allows us to estimate the false alert burden under realistic deployment conditions.

We acknowledge that stable COPD is not equivalent to a healthy physiological baseline and that subclinical deterioration or unreported events may exist during these periods. This limitation is inherent to real-world RPM datasets and is shared by most longitudinal exacerbation-prediction studies. Future work including healthy subjects could help characterize absolute baseline variability, but would not change the intended clinical use or evaluation framework of the proposed score.

We updated the Methods section to clarify the intended clinical use of the score and to justify the definition of control periods as clinically stable COPD intervals, including the temporal buffers applied around AECOPD events to reduce physiological contamination.

109 - 110 : Is "machine learning" correct here, or are these just signal processing methods. I think it's the latter, which is actually preferable (since then no training data is required) but it should be clarified.

We agree that describing the score as a “machine learning” method could be misleading. While the score uses Gaussian processes, a classical machine learning tool, these are applied in an unsupervised to model and process physiological time-series data, and not to learn from outcome labels (AECOPD events) to perform classification. The score itself applies statistical formulas to provide a risk score of exacerbations. As such, the BVS3 score can indeed be seen as an unsupervised statistical model rather than a machine learning one.

To avoid potential confusion, we have revised the 109-110 lines in the Methods section and revised the title (as well as the body) of the manuscript to:

“Short-term prediction of COPD exacerbations based on wearable vital signs monitoring”.

** 151 : The Rome protocol used 14 days, implying that a 13-day prior warning is not dissociated from the AECOPD event. So 14 days is the implicit default choice. The question arises, why was this not used in this study, and was 10 days chosen post-hoc since it gave the sharpest results?

Thank you for this comment. The Rome proposal indicates that the time from first onset of worsening respiratory symptoms to a full AECOPD ranges from 0–5 days in 90% of patients, with an upper biological limit of 14 days. Importantly, this consensus defines the maximum biological time frame over which an AECOPD may develop, but it does not prescribe an optimal prediction horizon for predicting AECOPD. These represent distinct methodological and clinical objectives.

In the field of short-term AECOPD prediction, prior studies have typically used 1 day, 3 days, 7 days or 14-day prediction horizon. This information has been added in the Discussion section in the new table comparing prior work.

While a 14-day window is biologically plausible, it corresponds to the upper limit of AECOPD development and may include events in which early alerts are temporally distant from the clinically manifest exacerbation, thereby reducing clinical actionability and weakening temporal attribution for a proportion of events.

In our pilot study, detections occurred as early as 8 days prior to AECOPD onset, indicating that a 7-day window was insufficient to capture early signals in a meaningful number of cases.

On this basis, a 10-day window was defined a priori as a compromise between early detection and clinical relevance, informed by pilot observations and existing literature, and not selected post-hoc based on performance optimization.

To address this concern further, we have clarified this rationale in the Methods section and added a sensitivity analysis comparing 7-, 10-, and 14-day windows in the Supplementary Materials. We found no statistically significant difference over the score performance between the three windows.

156, 160 : Is "30" a typo? Fig 2 indicates that this should be "20". Also, based on line 160, the period 20 - 30 days prior is not accounted for. The "dismissed period" choice makes good sense.

Thank you for highlighting this point. The “30 days” value is not a typo. Control periods in exacerbating patients were defined as 10-day windows beginning at least 30 days before or 30 days after the AECOPD event, which includes the −30 to −20 day window shown in Fig. 2. We have revised the Methods section to clarify these definitions.

** 164 : what is the clinical basis for 85% specificity? This results in a 20:1 ratio of false alarms to true alarms in this cohort.

Previous studies of remote patient monitoring systems suggest that an alert frequency of approximately five to ten alerts per patient per year is acceptable for healthcare professionals, whereas higher alert rates have been associated with reduced adoption. In the present study, we therefore defined a maximum acceptable alert burden of approximately six alerts per patient per year as the target operating point for short-term AECOPD prediction.

To achieve this, we report the performance of the BVS3 score at a fixed false-positive rate of 15%, corresponding to approximately six alerts per patient per year when using a 10-day prediction horizon. The manuscript was updated to include a rationale describing the choice of this operating point.

We also added a summary analysis of BVS3 false-positive alerts in the Supplementary Materials to contextualize their clinical relevance. Although the operating point selected for BVS3 yields a relatively high number of false positives, some of these alerts appear to be associated with physiologic changes consistent with clinically relevant events for pulmonologists, and in some cases may correspond to unlabeled exacerbations.

** 169 - 179 : The Rome protocol specifies "resting" vitals. However, the mean and std dev appear to be over all times. Would it be more appropriate to (a) use a Mahalanobis distance based on the distribution of resting vital sign values (mean and std dev will be lower than for all data points); and (b) restrict anomaly detection to just resting periods (based on activity detection data), to conform to the Rome protocol and avoid false alarms due to physical activity? This is a nontrivial issue, because activity could generate many false alarms. That is, your specificity might get much better at no cost to sensitivity if you restrict to resting periods.

Thank you for this comment. Analyzing biomarkers specifically during resting periods is challenging because no validated rest or sleep detection model was available on device, and developing such a model felt non-trivial. In the current analysis, outlier removal partially mitigates activity-related effects by suppressing values that are locally inconsistent with each patient’s distribution. In addition, we used long baseline windows and temporal filtering to smooth the time series and approximate underlying resting vital sign trends. That said, activity-informed normalization represents a promising extension of this work and will be explored in future studies.

Regarding the Mahalanobis distance, we agree that a multivariate formulation could better capture correlations and variability across patients’ vital sign distributions, and we plan to investigate this approach in future work.

** 168 ff : Can the BSV distinguish moderate vs severe AECOPD, or is it purely binary? What about mild cases? What are the clinical implications of this?

The BVS3 score is a continuous measure ranging from 0 to 10. In this study we explicitly present sensitivity at 15% FPR rate, corresponding to a threshold value of 3 on the BVS3 score. Alternative thresholds can be applied, for example a threshold of approximately 5 would correspond to a 5% false-positive rate, allowing flexibility in tuning sensitivity versus alert burden.

The BVS3 score has not been evaluated for mild exacerbations, which were outside the scope of this study, as mild events are managed by patients themselves.

Regarding moderate versus severe AECOPD, the score currently does not include a validated stratification by severity. While the level of BVS3 is correlated with the amplitude of vital-sign anomalies, we have insufficient hospitalization data to formally evaluate associations with event severity. As shown in Figure 4, although the median BVS3 score tends to be higher for severe events compared with moderate events, this difference did not reach statistical significance in our cohort.

Alerts based on a BVS3 threshold should therefore be interpreted as a binary alerting tool for clinically actionable exacerbations (moderate or severe) rather than a tool for discriminating severity or for detecting mild events. Threshold selection can be adjusted to balance sensitivity and alert burden according to clinical priorities, but current evidence does not support using BVS3 for fine-grained severity stratification.

183 : Were there any rejection criteria, eg for values that are simply unrealistic (eg zero motion, HR < 40, temperature < 34, etc)?

The device used to collect vital signs is CE-marked Class IIa and has the following validated operating ranges: heart rate 35–180 bpm, respiratory rate 10–50 breaths/min, and oxygen saturation ≥70%. Values outside these ranges are not reported by the device.

Additionally, outlier filtering was performed using a threshold on the median absolute deviation (MAD), which identifies extreme values relative to the observed data distribution without relying on predefined cutoffs. We have clarified this procedure in the Methods section.

** 189 : "with each contributing equally". This is very surprising, because there is no a priori reason (that I know of) that these symptoms should have equal salience. Is there a reason for equal weights vs calibrating individual weights on a test set?

Thank you for this important comment. The equal weighting was a deliberate methodological choice consistent with the unsupervised nature and objective of our approach.

The BVS3 algorithm does not use outcome labels and is not trained to predict AECOPD events; therefore, there is no supervised criterion available to calibrate or optimize individual weights on a test set. Instead, each vital sign is first normalized using Z-scores, which places all signals on a comparable scale and allows deviations to be interpreted as relative anomalies with respect to each patient’s own baseline.

The goal of the composite score is not to model the relative clinical importance of each vital sign, but to identify time periods during which multiple vital signs exhibit simultaneous abnormal behavior. Assigning equal weights ensures that the composite score reflects concurrence of anomalies rather than privileging one signal a priori without outcome-based justification.

We agree that alternative weighting strategies could be explored in a supervised or semi-supervised framework, or informed by external clinical priors. However, introducing unequal weights in the present unsupervised setting would require assumptions about relative salience that cannot be empirically validated within our study design.

We updated the Methods section to explain this design choice.

192 - 193 : Perhaps take a moment to walk the reader through this equation, so that the reader is not bogged down unnecessarily.

Thank you for this suggestion. We have revised the manuscript to provide a concise walkthrough of the BVS3 score calculation to help the reader understand how the composite score reflects concurrent anomalies.

** 193 : I do not understand the "2" in eqn 3. It looks like you are capturing the probability of both tails, but it is unclear why this is desirable. The adverse conditions are in one direction only (ie elevated HR, BR, 100 - SpO2). So would a one-sided tail would be appropriate? On that topic, would a one-sided std dev, calculated only on points greater than the mean, be a more accurate parameter?

Thank you for raising this point. To clarify, all Z-scores are oriented so that higher values indicate adverse conditions (elevated HR and BR, low SpO₂). The factor of 2 in the equation is not related to two-sided tail probabilities; it is merely a scaling factor applied to the average Z-score before the survival function and log transformation. Changing this factor would only scale the resulting score but does not alter the one-sided nature of the calculation. Thus, the composite score reflects deviations in the clinically relevant direction only.

Regarding the use of a one-sided standard deviation calculated only on points above the mean, our Z-scores are computed using the standard deviation of all baseline values. This approach provides consistent normalization across patients and time points, ensuring comparability of relative anomalies. While a one-sided standard deviation could, in principle, slightly change the scale of the Z-scores, we expect it would not meaningfully affect the detection of concurrent anomalies, which is the primary goal of the composite score.

** 197 : "threshold value of 3": on what data was this determined? It must be different data from this cohort, or else the cohort needs to have a k-fold split (stratified by patient) to make the threshold selection independent of the results. Also (as noted above) what is the clinical justification for 85% specificity?

Thank you for pointing out the need for clarification.

The threshold was not calibrated or optimized using a separate dataset. Instead, discrimination of case and control periods by the BVS3 score was first evaluated across the full range of decision thresholds using ROC analysis on the study dataset. A target specificity of 85% (false positive rate of 15%) was defined a priori based on anticipated clinical deployment constraints, corresponding to an acceptable alert burden in routine use.

The numeric threshold value (BVS3 = 3) corresponds to the score yielding this predefined operating point on the ROC curve.

Importantly, the alert threshold was not selected to maximize outcome-based performance metrics, and no data-driven optimization or calibration was performed. Because threshold selection was based solely on a fixed specificity criterion applied after ROC construction, this approach minimizes overfitting concerns associated with threshold tuning. We have updated the Methods section accordingly to clarify this point.

213 : are there any patient characteristics that would affect PPG readings, eg skin color?

The device used to collect vital signs complies with ISO 80601-2-61, which requires equivalent performance across different skin tones.

213 (and "limitations" also): How does the overall sickness of this cohort relate to the expected cohorts in the envisioned use cases? For implications, see for example Kendall "Whom tuberculosis tests detect and why it matters" 2025.

After reading the referenced article, we agree that performance across GOLD severity stages should ideally be balanced. To address this point, we note that the cohort was recruited without inclusion criteria related to disease severity and was therefore intended to reflect the distribution of GOLD stages typically observed in the general COPD population. Nevertheless, we acknowledge that the study was conducted at a single French hospital, which may limit generalizability. As such, a larger multicentric study would be more appropriate to robustly assess performance across all GOLD stages, and this limitation is explicitly discussed in the manuscript.

251 : As noted above, 85% specificity gives a very high number of false alarms. For a way to assess ROC curves in this context, see Delahunt "Metrics to guide development of ml algorithms for malaria diagnosis" 2024.

You are right to point out that the AUC of ROC curves alone is not an optimal metric for evaluating the performance of a clinical algorithm. For this reason, we focused on a prespecified operating point of 15% FPR, which we acknowledge is debatable, as it may appear to place a high burden on clinical teams, but we believe represents a reasonable compromise, as discussed in our response to comment 197.

302 : "aligned with the 2025 GOLD recommendations [24]": these come directly from the Rome protocols [5]. Perhaps note this for clarity.

We have revised the manuscript sentence to clarify this lineage and give proper attribution to the original source.

310 : Perhaps note that each patient needs to have a baseline distribution for each vital sign to use the method.

Thank you for raising this point. The computation of patient-specific baselines is integrated within the BVS3 method and typically requires 3–5 days of data, depending on the number of measurements per day. Because this baseline computation is built into the algorithm, the BVS3 score remains robust and directly transferable across different monitoring systems, maintaining the device-agnostic design described in the manuscript. We modified the manuscript accordingly.

** 325 : "high performance and usability for real-time care": this is overly optimistic, unless the results are adequate for concrete clinical use cases. Eg it must have sufficient sensitivity and few enough false alarms; the cohort of this study is sufficiently representative of expected cohorts in known use cases.

To address the concern of over-optimism, we have updated the text to describe the BVS3 score as demonstrating “good preliminary performance and usability” rather than “high performance.”

To contextualize these results, we added a comparative table summarizing prior published work on AECOPD short-term prediction using similar metrics (AUC and sensitivity at ~15% FPR). As shown in the table, the BVS3 score achieves AUC 0.88 and sensitivity 74% at 15% FPR, outperforming most prior approaches based on symptom questionnaires, wearables, or EHR-derived data. Notably, many previous studies either relied on self-reported exacerbations, had unclear case definitions, or had limited validation cohorts, which can inflate apparent performance or limit generalizability.

While we acknowledge that the present study is monocentric and further multicentric prospective validation is ongoing, the data indicate that the BVS3 score achieves robust preliminary performance for clinically actionable moderate and severe exacerbations and compares favorably with prior work in both sensitivity and specificity.

343 - 344: "the very high specificity": this is baffling. It does not agree with the plots, which show quite low specificity at moderate sensitivities.

You are right, thank you for pointing that out. This is a typo as we were referencing the values of *sensibility* of the most severe exacerbations at 15% specificity. However, the number of severe exacerbations (n=7) is too low for a generalization; we have modified the manuscript accordingly to be clearer and more cautious.
